# Reciprocal control of cell proliferation and migration

**DOI:** 10.1186/1478-811X-8-20

**Published:** 2010-09-07

**Authors:** Alina De Donatis, Francesco Ranaldi, Paolo Cirri

**Affiliations:** 1 Dipartimento di Scienze Biochimiche, Università di Firenze, Viale Morgagni 50, 50134 Firenze, Italy

## Abstract

In adult tissue the quiescent state of a single cell is maintained by the steady state conditions of its own microenvironment for what concern both cell-cell as well as cell-ECM interaction and soluble factors concentration. Physiological or pathological conditions can alter this quiescent state through an imbalance of both soluble and insoluble factors that can trigger a cellular phenotypic response. The kind of cellular response depends by many factors but one of the most important is the concentration of soluble cytokines sensed by the target cell. In addition, due to the intrinsic plasticity of many cellular types, every single cell is able, in response to the same stimulus, to rapidly switch phenotype supporting minimal changes of microenviromental cytokines concentration. Wound healing is a typical condition in which epithelial, endothelial as well as mesenchymal cells are firstly subjected to activation of their motility in order to repopulate the damaged region and then they show a strong proliferative response in order to successfully complete the wound repair process. This schema constitute the *leitmotif *of many other physiological or pathological conditions such as development vasculogenesis/angiogenesis as well as cancer outgrowth and metastasis.

Our review focuses on the molecular mechanisms that control the starting and, eventually, the switching of cellular phenotypic outcome in response to changes in the symmetry of the extracellular environment.

## Introduction

Receptor tyrosine kinase (RTKs) is a very important class of transmembrane proteins whose function is to sense and transduce extracellular environmental changes. RTKs regulate many aspects of cellular physiology both during development and in adult life, such as cell proliferation, migration and differentiation [1]. Ligand binding mediated activation of RTKs consists in the receptor dimerization and activation of its intrinsic tyrosine kinase activity that leads to transphosphorylation on specific tyrosine residues that act as docking sites for intracellular signaling proteins [2]. The recruitment of these proteins leads to the activation of many signaling pathways, including ERK1/2, phosphatidylinositol 3-phosphate kinase (PI-3K), phospholipase C-γ (PLC-γ), the Src family of tyrosine kinases, the SHP-2 tyrosine phosphatase and the signal transducers and activators of transcription (STATS) whose function is to transduce the activation signals to the nucleus eliciting the corresponding transcriptional response.

The pleiotropic functions elicited by this receptor's family raise many questions about how their specificity of action could be achieved. In fact, it is hardly conceivable that the activation of a single type of receptor could exert so different, if not mutually exclusive, physiological role using the same transduction mechanisms and the same intracellular signaling modules. The most obvious explanation is that different cell types and/or different differentiation stage for a given cell could respond differently upon stimulation with the same cytokine having a substantially different protein expression patterns and, consequently, different intracellular signaling modules and/or regulatory pathways. More difficult, in our opinion, is to explain how a single RTK in a given cell type can induce different phenotypic response. In fact, there are many physiological or pathological condition (i.e. in wound repair, during development, in angiogenesis, during metastatic process, etc.) in which cells have to shift their phenotypic output from migratory to proliferative one in response to the same kind of stimulus. Wound healing, for example, is a dynamic and complex process that restore tissue homeostasis after an injury, which requires cell migration of different kind of cells (i.e. epithelial cell, fibroblast etc) as well as cell proliferation of the same cellular types. In a first phase of wound repair process, many cytokines, such as interleukin 1 (IL-1), epidermal growth factor (EGF), platelet-derived growth factor (PDGF), vascular endothelial growth factor (VEGF) and transforming growth factor-beta (TGF-β), are secreted by keratinocytes and platelets in the wound area and activate inflammatory response and the recruitment of immune system cells. In a later stage, the presence within the wound area of a similar pattern of cytokines, produced for an important part by macrophages, contribute to reconstruct the damaged tissue, through the induction of the migration and proliferation of different cells that lead to angiogenesis, granulation tissue formation and epithelization. For the correct completion of this process, many kind of cells (fibroblasts, endothelial as well as epithelial cells) have to dynamically change their behavior even in response of the same kind of stimulus. PDGF for example is a cytokine that possesses both chemotactic and pro-mitogenic action on fibroblast, but for a single cell at one time these two cellular response are mutually exclusive.

Hence the questions are: i) which kind of extracellular event or condition can induce a given cellular phenotype and not the other; ii) how this extracellular signal is correctly transduced within the cell conserving the specificity; iii) which are the intracellular effectors, mechanisms and timing events that allow the correct execution of the program.

Remaining in the wound repair context the key decider of cell behavior is the cytokine concentration sensed by the cell. In fact we showed [3] that a fibroblast can proliferate or migrate in relation to the environmental PDGF concentration. Cytokines produced by cells present in the wound area (macrophages for example) give raise to a gradient that act as a chemoattractant factor for more distant cells that sense a relatively low ligand concentration and their phenotypic response consists in cell migration along the gradient. When migrating cells arrive at a point where the cytokine concentration reaches a precise threshold they switch from a migrating phenotype to a proliferating one, leading, in the case of wound healing example, to an efficient tissue repairing. In "in vivo" conditions the establishing and the dynamically maintaining of the cytokine gradient is favored by the viscosity of the ECM matrix and by the glycosylated form of the cytokines themselves. A "stable" gradient could be essential for inducing a correct behavior of target cell. The most important cellular sensors of chemoattractant gradient are the RTKs that act not only as signal transducers but also as a relay that drives cellular decision about migration/proliferation switch. Two fundamental differences distinguish cell proliferation and cell migration: i) cell division is an irreversible process (it is started by a single and rapid event, i.e. ligand stimulation of a growth factor receptor and the cell is immediately committed to that long lasting process without the possibility of changing the actual cellular programme), while migration is a reversible one, that is the signaling system that sustain migration can quickly be stopped, can be changed "on the run" the direction and the speed of movement and even there can be a switch in the phenotypic response [4]; ii) cell migration is mediated by a non-symmetric cell polarization while cell proliferation is not. Essentially, the starting and the driving event that is responsible of the choice between this opposite output (the RTKs) must be, in turn, able to start a cyclic and reversible signal or a unique and irreversible one.

De donatis *et al. *[3] showed that relatively low concentration of PDGF are able to induce exclusively a motile phenotype by activating only a limited subset of the well known PDGF-R activatory pathways [5,6], in particular are selectively activated pathways involved in cytoskeleton dynamic remodeling such as Rho, Rac and FAK. In addition, low growth factors doses induce exclusively clathrin-mediated endocytosis (CME) in which most or the receptor is not degraded [3,7], but it is recycled back to the plasma membrane where it can act as a sensor for driving directional cell movements. In a very important work Jékely et al. [8], demonstrate that, in drosophila model, RTKs elicit polarized signaling within a cell being localized in the leading edge of the migrating cell. They also showed that receptor endocytosis is required for this kind of signaling restriction and that Cbl and Spring are the two signaling proteins that mediate this event. Cbl is a protein involved in both ubiquitination and lysosomal degradation of many RTKs and in the regulation of endocytosis [9], while the mammalian counterpart of Sprint, called RIN1, displays Ras-activated Rab5 guanine nucleotide exchange factor (GEF) activity and is as well involved in EGF-R endocytosis [10]. In this context, clathrin-mediated receptor endocytosis is necessary for keeping active signaling complexes localized near the plasmamembrane preventing signaling from becoming uniform within the cell and therefore uninformative about the cytokine gradient.

The localized RTKs activation lead, in turn, to the localized activation of the downstream signaling modules that start and maintain cellular polarization acting on the structure and function of cellular cytoskeleton. One of the master regulator of leading edge formation is FAK. In a very recent work [11] Long et al. have identified in an alternate-spliced isoform of the steroid receptor coactivator-3 (SRC-3Delta4) the linker between EGFR activation, FAK and enhanced cell migration. SRC-3Delta4, in consequence of EGFR stimulation, becomes phosphorylated by PAK1, translocates to plasmamembrane where acts as a bridge between EGFR and FAK leading to the activation of FAK itself. FAK phosphorylation on tyrosine 397 creates high affinity binding sites for the SRC homology 2 (SH2) domain of the tyrosine kinase Src and for several other proteins [12]. The FAK-Src association leads to phosphorylation of FAK by Src on tyrosine 576 and 577 which fully activate FAK [13] and on tyrosine 925 which is critical for FAK promotion of cell migration [14]. One of the main target of FAK-Src activation is p130Cas [15] that behaves as a scaffolding protein and recruits many downstream signaling proteins such as Rap1 and Rac [16]. The localized formation of FAK protein complexes gives origin to nascent focal adhesion sites at cellular leading edge, a process that facilitates cell polarization in the direction of cell migration.

**Figure 1:**
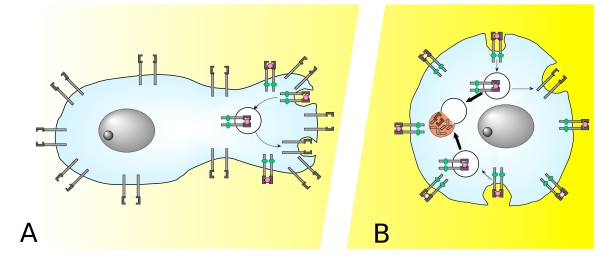
**Background color represents the extracellular ligand gradient**. **A) **Cell far away from the gradient source senses a relatively low ligand concentration. The limited and localized RTK activation induce in turn a primitive formation of submembrane signaling complex that starts organizing focal adhesion structure and induce cellular cytoskeleton remodeling, leading to progressive cellular polarization along the gradient. Local RTK chlatrin-mediated endocytosis of active receptors is the event that promotes and maintains the cyclic process of directional cell migration. **B) **When migrating cell arrives in a area in which cytokine concentration reaches the "mitotic" threshold the number of activated RTKs increases. Hence, the environmental relative loss of asymmetry is reflected by a not localized RTKs activation that, in turn, induce the loss of cellular asymmetry that stops the migrating process. In addition, the massive RTKs activation leads to triggering of an additional endocytotic route (RME) that relocates RTKs prevalently to the endosomal compartment changing radically the active intracellular signaling modules. The RTKs endosomal signaling is responsible for the activation of the vast transcriptional programme that ultimately leads to mitosis.

Hence, in migrating conditions, RTKs is subjected to a cyclic process of CME-mediated internalization and strictly localized re-uptake to the plasmamembrane that in turn directionally guide the cyclic process of cell migration maintaining the asymmetric information of the environment given by the ligand gradient (Figure 1A). Of note, the activated RTK endocytosis may serve also as a mechanism for detach the ligand from the internalized receptors before they recycle back to the membrane, since only free receptors can act as an active sensors for directional migration.

The directional cell migration along an increasing ligand gradient take places until migrating cells reach a zone in which they start dividing as a results of the gain of the appropriate ligand threshold that commits cells to mitosis. In that moment cells switch from reversible and asymmetric phenotype (migration) in a irreversible and symmetric one (mitosis).

This threshold could be represented by the ligand concentration at which there is no more asymmetry in the ligand distribution in the environment around the cell and consequently no directional indication. In parallel to the loss of asymmetry of the extracellular milieu a much greater number of receptor become activated by ligand with respect to migrating conditions [3] and, likely, their distribution along the plasmamembrane become homogeneous rather then localized (Figure 1B). In addition, high level of RTKs engagement by ligand induce RTKs internalization also through Rafts/Caveolin-mediated endocytosis (RME) [7]. It is well established that RTKs endocytosis, far for being exclusively a pathway for receptor downregulation, play an important role in signal transduction [17,18]. In particular, for what PDGF-R concerns, Wang Y. et al. [19] showed that endosomal PDGF-R signaling is sufficient to activate the major signaling pathways that allow cell proliferation. The relocation of the receptor from plasmacellular or sub-plasmacellular to "cytosolic" site changes the intracellular signaling proteins recruited by the receptor itself and, therefore, this event leads to the activation of pro-mitogenic signaling modules (MAPK, PI-3K etc) [3,19], in the meanwhile the recycle rate of PDGF-R to the cell surface is reduced and the receptor is addressed to late endosome/lysosomal compartment for degradation, consistently with the fact that now the cell is committed irreversibly to mitosis and RTK has ended its function for the remaining cell cycle completion time.

## Conclusions

A clear understanding of the regulatory mechanisms that represent the molecular basis of cellular behavior in response to variation of extracellular environment is essential to elucidate many physiological as well as pathological processes.

The evidences till now available have clarified some aspects of the very complex problem of cell signal integration, differential activation of signaling modules and intracellular compartmentalization of active complex that constitutes the inner causes of cellular response to changes in extracellular environment.

## Competing interests

The authors declare that they have no competing interests.

## Authors' contributions

ADD wrote the initial draft of the manuscript, PC revised and completed it. FR prepared the figure and gave important intellectual contribution. All authors have given final approval of the version to be published.
